# Lumbar Puncture

**Published:** 1906-09-29

**Authors:** 


### LUMBAR PUNCTURE.

Lumbar puncture has been used now for many years in hospital work; its technique and in some cases the assistance required has prevented the general practitioner from having recourse to it; in most cases it is quite easy to perform, and with care has practically no danger. The patient should be lying down, and the fluid should be let run out, and not aspirated.

In diagnosis very little importance can be attached to a negative result, but in cases in which a cerebral condition is obviously present there is much to be learnt by a bacteriological examination, negative or positive, taking also into consideration the colour of the fluid, the tension at which it escapes, and the variety of the cells found in it (cyto-diagnosis).

Cryoscopy and hæmolysis are more complicated and less trustworthy.

In cases of head injury the presence of blood is of much value in diagnosis, and when compression is present the lowering of tension by letting some of the fluid run out relieves symptoms and helps towards a cure.

Its value in the relief of headaches and hydrocephalus is occasionally more than temporary, and it has been found of use in cerebro-spinal meningitis, uræmia and spastic torticollis.

Its use for the introduction of anæsthetics is under discussion, and is not without risk; still, recent literature has shown that there are certain selected cases in which it will continue to be of use.

Lastly, it has achieved some success in washing out the whole length of the spinal cord by a continuous low-pressure stream of saline from a small cannula introduced at the base of the brain.

Although it is not to be expected that the practitioner should be able to follow the ceaseless changes brought about in pathology by modern research and altering nomenclature; yet he wishes to know the main facts as soon as they have made a definite advance since his school days. Few journals can now be read without the word endothelioma being met with. It is neither a new word nor tumour, but its place is now defined in classification, and a few words about it will not be out of place.

This new growth for the most part is malignant, but some of them are definitely encapsuled and microscopic examination by a skilled eye can more or less determine their innocency. They arise from the endothelium lining lymphatic spaces, and have different names and appearances in various situations. Their commonest sites are the testis, the salivary glands, the cheeks, lips, and palate, and the long bones; they are also found arising from the peritoneum, pleura, and meninges.

The growth consists of large polygonal cells arranged in alveoli, with a varying amount of intervening connective tissue, which often gives them a superficial resemblance to the carcinomata.

The psammoma of the brain is a sub-division of these tumours, as also the cylindroma and myxosarcoma, the latter variety being due to a change in the matrix. Many of them have been hitherto called lympho-sarcoma. Perithelioma is a class in which the cells are grouped one or two layers thick round a capillary, and arranged as is a vascular network; these are most common in connection with the pia mater.

It is quite possible that the much-discussed tumour, variously known as deciduoma malignum, chorio-epithelioma, or syncythioma, will eventually find its place amongst the endotheliomata. In treatment and in their clinical course they do not differ materially from the other varieties of the great class sarcomata, to which they all belong.

